# Warburg effect(s)—a biographical sketch of Otto Warburg and his impacts on tumor metabolism

**DOI:** 10.1186/s40170-016-0145-9

**Published:** 2016-03-08

**Authors:** Angela M. Otto

**Affiliations:** Institute of Medical Engineering (IMETUM), Technische Universitaet Muenchen, Boltzmannstr. 11, D-85748 Garching, Germany

**Keywords:** Biography, Warburg effect, Glycolysis, Respiration, NAD(P)H, Tumor cells, Spectroscopy, Manometer, Hypothesis, Nobel prize

## Abstract

Virtually everyone working in cancer research is familiar with the “Warburg effect”, i.e., anaerobic glycolysis in the presence of oxygen in tumor cells. However, few people nowadays are aware of what lead Otto Warburg to the discovery of this observation and how his other scientific contributions are seminal to our present knowledge of metabolic and energetic processes in cells. Since science is a human endeavor, and a scientist is imbedded in a network of social and academic contacts, it is worth taking a glimpse into the biography of Otto Warburg to illustrate some of these influences and the historical landmarks in his life. His creative and innovative thinking and his experimental virtuosity set the framework for his scientific achievements, which were pioneering not only for cancer research. Here, I shall allude to the prestigious family background in imperial Germany; his relationships to Einstein, Meyerhof, Krebs, and other Nobel and notable scientists; his innovative technical developments and their applications in the advancement of biomedical sciences, including the manometer, tissue slicing, and cell cultivation. The latter were experimental prerequisites for the first metabolic measurements with tumor cells in the 1920s. In the 1930s–1940s, he improved spectrophotometry for chemical analysis and developed the optical tests for measuring activities of glycolytic enzymes. Warburg’s reputation brought him invitations to the USA and contacts with the Rockefeller Foundation; he received the Nobel Prize in 1931. World politics and world wars heavily affected Warburg’s scientific survival in Berlin. But, after his second postwar recovery, Warburg’s drive for unraveling the energetic processes of life, both in plants and in tumor cells, continued until his death in 1970. The legacy of Otto Warburg is not only the Warburg effect, but also the identification of the “respiratory ferment” and hydrogen-transferring cofactors and the isolation of glycolytic enzymes. His hypothesis of respiratory damage being the cause of cancer remains to be a provocative scientific issue, along with its implications for cancer treatment and prevention. Warburg is therefore still stimulating our thinking, as documented in a soaring increase in publications citing his name in the context of tumor metabolism.

## Background

**Fig. 1:**
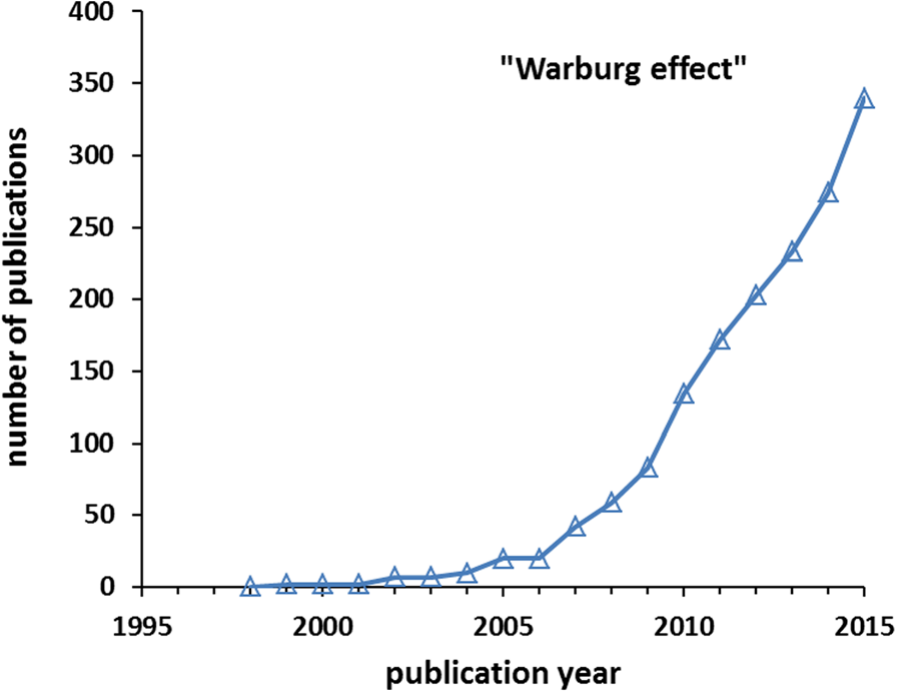
Number of publications per year found in the Web of Science with the search words “Warburg effect” AND (cancer OR tumor)

The work of Otto Heinrich Warburg has been having a profound influence on the way we think about tumor metabolism. Particularly, in the field of cancer research, we are using the term “Warburg effect” to denote a metabolic phenotype typical of many tumor cells, namely the high activity of anaerobic glycolysis, i.e., production of lactic acid, even in the presence of sufficient oxygen. Originally however, this term was first used in 1962 in plant physiology to denote the inhibition of CO_2_-fixation by oxygen in photosynthesis [1]. It was then Efraim Racker who in 1972 in his publication on the bioenergetics of tumor growth coined the presence of high aerobic glycolysis in tumors as the Warburg effect [2]. Yet, this term was not generally used in this context until about 30 years later. Especially during the last 15 years, the number of publications using the term Warburg effect in cancer research has risen quasi exponentially, a sign of the renaissance of tumor metabolism (Fig. 1).

But, who was the man behind the Warburg effect? What were his scientific achievements, and which of these lead to the discovery of this “effect” almost a century ago? And, in which scientific context does his work appear today?

## An illustrious personal background

To better appreciate Warburg’s ground breaking experimental achievements and his way of thinking, it is worthwhile going into his personal history, recounting which influences his family background as well as his social and scientific environment had on him. Otto Heinrich Warburg (not to be confused with his uncle Otto Warburg, a botanist) was born on October 8, 1883 in Freiburg in Breisgau, a city with a traditional university in southwestern Germany. His father, Emil Warburg, at that time an eminent Professor in Physics at the University of Freiburg, belonged to a widespread Jewish family (which in the sixteenth century had settled in the German town of Warburg), but he had become a Protestant. Otto’s mother, a sociable and resolute person, came from a South German family of military and governmental officials. Therefore, his parents were predestined to educate him and his three sisters in an intellectually and culturally stimulating environment with a gracious lifestyle typical of the academic class during the imperial reign of Germany. In 1896, the family moved to Berlin, where Emil Warburg had become Director of the Institute of Physics at the University of Berlin. Through his membership in the prestigious Prussian Academy of Sciences, he was in close contact with scientific colleagues such as Albert Einstein and Max Planck. The house of the Warburgs was the site of vibrant social evenings, where Einstein played the violin, Planck played the piano and other colleagues such as J. H. van’t Hoff and Walter Nernst contributed to the musical, literary, and philosophical entertainment. No doubt, these guests seeded and fostered Otto’s interest in natural science and molded his personality. His contemporaries later would not only describe him as a charming, humorous, and generous person, but also as being eccentric as well as polemic and vindictive toward his opponents, as lacking modesty and not accepting any criticisms of his ideas. Certainly, his extraordinary intelligence, his disciplined style of working and living, and his sharp mind were outstanding characteristics as a scientist (biographical notes in this article were taken from *Krebs*, *Schmid* [3], and *Werner* [4])*.*

Otto Warburg decided to study chemistry and left Berlin in 1901 to enroll at the University of Freiburg, from where he returned in 1903 to become a student of Emil Fischer. Fischer had just received the Nobel Prize the year before for the synthesis of sugars and purines; he was a demanding teacher, requiring precision in experiments and thinking, and perseverance in science. In his lab, Warburg worked on the chemistry of glycine, alanine, and leucine, for which he received his doctoral degree in 1906. With this scientific foundation—and the advice of his mother not to marry (marriage being incompatible with the life of a scientist)—Otto Warburg was well on his way to a promising scientific career.

But, Warburg first wanted to know more about the processes in life and thus went to the University of Heidelberg to study medicine with Ludolf Krehl, Professor of Pathophysiology. There, he also got acquainted with Julian Huxley, an evolutionary biologist, with Viktor von Weizsäcker, a founder of psychosomatic medicine, as well as Archibald Vivian Hill and Otto Meyerhof. Warburg wanted to apply the concepts and methods he had acquired in chemistry and physics to understand the energetic processes of life. He started experimenting with different types of cells: bacteria, yeast, and red blood cells. During the summers of 1908 to 1911, often along with Meyerhof, he visited the Marine Station in Naples (Italy), where he made his first remarkable observations with sea urchin eggs; he found that oxygen consumption was increased six- to sevenfold after the eggs had been fertilized and was strongly inhibited by cyanide and narcotics. This work converged into a second doctoral thesis in 1911 on the oxidation of live cells performed with sea urchin eggs as a model system [4]. In 1913, he acquired the qualifications for a professorship in physiology (“Habilitation”) on the topic of energy-delivering reactions in live cells.

## Establishing a scientific career

In 1913, Warburg decided to return to Berlin, where the Kaiser Wilhelm Institute (KWI) had been founded and of which his previous mentor Emil Fischer was the Vice-President. With the support of such notable scientists as Paul Ehrlich (who recognized the importance of basic cancer research), Theodor Boveri (who had hypothesized that cancer was due to chromosomal damage) and Jacob Loeb (a friend of the Warburg family and a German emigrant working at the Rockefeller Institute in New York on sea urchin parthenogenesis), Otto Warburg came to be a member of the KWI. He got a position as a principal investigator at the new Institute of Biology—with the freedom to choose his research topic. Since he disliked teaching obligations as well as working in the industry, this was the ideal position for him.

At the outbreak of the First World War in 1914, Warburg felt a traditional and national obligation to serve the country, and he volunteered—much to the dismay of his mother. He was installed as a physician in an elite cavalry unit. In 1918, after having been wounded, Otto’s mother now insisted that her son could do more good for his country by going back to science rather than to the battle-field. So, she wrote to Albert Einstein to support her mission in convincing the ministry—and her son. And indeed, upon receiving a letter from Einstein, Warburg returned to Berlin and to the lab bench. Moreover, to relieve him of organizing his daily life at home, Otto’s mother now organized a personal aid, a reputable young man named Jacob Heiss. Eventually, he would also be Warburg's secretary, doing the unavoidable administrative work of the institute and becoming a loyal friend accompanying Warburg for the rest of his life.

In spite of the thwarted financial state after the First World War, Warburg managed to continue his research. The German government had established funds for supporting research of scientists in precarious economic situations. Warburg asked for such funding on the telephone, but a written application was required. So Warburg dictated his text to a secretary which had been hired for this purpose; the application consisted of a single page and had the following content:

- Dr. Otto Warburg.
  - ᅟ
    - Application
  - I need ten thousand Marks.
    - *Otto Warburg*

The money was granted.

Of the long scientific career that followed, only a few highlights can be alluded to in this short summary of Otto Warburg’s life and work. His outstanding achievements evolved from the synthesis of excellent experimental training and innovative thinking, which circumvented around two basic questions. (1) What are the energetics of life? (2) Is there a quantitative difference in the metabolism of cancer cells and normal cells? For the experimental, i.e., technical setup, he recruited not academics with ambitions for their own career, but rather professionals trained in fine mechanics, capable of constructing the particular devices needed for investigations with tissue and cells; they were frequently coauthors of his publications. Otto Warburg profited from his father in having learned to work with a manometer, with which it was possible to measure in solution CO_2_ production and O_2_ consumption. By providing a temperature control and a shaking mechanism to ensure rapid diffusion in the medium, the device was modified to serve the specific requirements for measuring live tissues and cells. Since the tissue needed a standardized thickness, he calculated the number cell layers which allowed for sufficient diffusion of glucose and O_2_, and developed a mechanism for preparing tumor slices of <0.5 mm—a method still in use today. Moreover, by parallelizing the setup, it was possible to measure multiple samples at the same time. These technical innovations allowed Warburg and his coworkers to perform the pioneering experiments leading to metabolic concepts still being investigated today.

For the scientific part, Warburg had intelligent and motivated PhD students and postdocs (wissenschaftliche Assistenten). Warburg’s lab was open to visiting scientists, among them being David Keilin (who had discovered the cytochromes), Archibald Vivian Hill (working on O_2_-consumption in muscle), Hugo Theorell (a Swedish biochemist working on oxidizing enzymes), Fritz Albert Lipmann (discoverer of coenzyme A), and Severo Ochoa (a Spanish biochemist working on biosynthesis of nucleic acids) [4]. It is fortunate that Hans Krebs, who worked in Warburg’s lab from 1926 to 1930 and maintained a long standing relationship with him, recollected Warburg’s life in a biography illuminating his personality and scientific achievements [3].

## First observations on tumor metabolism

In 1923, Otto Meyerhof and Archibald V. Hill received the Nobel Prize for work on the energetics of muscle metabolism, in particular for the discovery of the relationship between oxygen consumption and lactic acid metabolism. In the same year, Otto Warburg and Seigo Minami published the first observations on changes in the metabolism of tumors [5]. They had observed that tumors acidified the Ringer solution (an isotonic salt solution, with 2.4 mM NaHCO_3_) when 13 mM glucose was added, as indicated by a change in the color of organic pH-indicators. In this acidified solution, lactic acid was chemically identified. To better quantify this phenomenon, Otto Warburg modified the Barcroft manometer to measure slices of a Flexner-Jobling rat hepatoma, which he had received from Rhoda Erdmann at the Rockefeller Institute. The amount of lactate produced was calculated from the increase in CO_2_-formation during a 30-min incubation period. Surprisingly, the tumor tissue had a 70-fold higher rate of lactate formation than the normal liver as well as kidney and heart tissue likewise tested. This is the observation that would more than 50 years later be referred to as the Warburg effect. Lactate production did not depend on the presence of oxygen. That had not been expected, since according to Pasteur, the presence of oxygen should have suppressed glycolysis. The fact that there appeared to be no direct relationship between respiration and glycolysis lead to the conclusion that in cancer cells, glycolysis was a reaction which could produce energy, independent of respiration (oxygen consumption). In other experiments with varying glucose and bicarbonate concentrations, it was shown that there was no generalizable difference in oxygen consumption between the tumor and the respective normal epithelial tissue [6]. In 1924, Warburg hypothesized that there was a defect in the relationship between glycolysis and respiration. Even though this observation was corroborated with other tumors by several contemporary scientists [7], the observation that oxygen could not suppress glycolysis prompted him to propose that a damage in respiration leads to carcinogenesis [8]. This came to be a highly controversial issue climaxing in his famous papers in Science in 1956 [9].

Testing the effects of other parameters, Warburg and coworkers changed the pH of the Ringer solution ranging from pH 7.83 to 6.66 using 1–15 % CO_2_-N_2_ gas mixtures, respectively. The rate of CO_2_-production (interpreted as glycolysis) increased with increasing alkaline pH. Moreover, a tenfold increase in bicarbonate concentration at a defined pH of 7.5 also increased CO_2_ production [10]. Warburg interpreted these conditions as being similar to those in blood passing through capillaries, leading at the same time to a modest acidification and to an increase in bicarbonate concentration. In the balance, glycolysis of the tissues would not change. On the other hand, other studies showed that in tissue homogenates, alkalinity increased with dedifferentiation and necrosis of tumors [11], suggesting that the tumor itself may have a different pH. However, the influence of pH on the growth of tumor cells appeared never to be of particular interest to Warburg, in spite of his interest in hydrogen-transferring systems such as the coenzymes NAPDH and NADH (see below), which lead to the characterization of the activity of most glycolytic enzymes in later years.

Warburg corroborated his in vitro results in rats having either a hepatoma or sarcoma, where he found a higher lactic acid content (chemically determined) in blood vessels leaving the tumor than in vessels entering the tumors [12]. Similar experiments had been performed by Carl and Gerty Cori [13], who also found different lactic acid levels in the blood of the two wings of same chicken: one with the implanted tumor and one without it. Warburg’s interpretation was that a lack of oxygen (hypoxia), along with an increase in lactic acid, favored the survival of tumors as opposed to normal cells, since the latter could not recruit their energy from anaerobic glycolysis. In other words, his hypothesis was that chronic hypoxia would damage respiration. The basis for this line of thinking was that, according to the Pasteur Effect, the presence of oxygen should (completely) suppress glycolysis. Since this was not the case in tumor cells, he concluded that there were “disturbances in the relationship between respiration and glycolysis” [10]. In 1930, Warburg reinforced his hypothesis in stating that anaerobic glycolysis of tumor cells is the result of respiratory damage (Schädigung) [14]. This issue was critically discussed by Dean Burk at a Cold Spring Harbor Symposium on Quantitative Biology in 1939, where he presented a collection of data from different tumors showing that tumor cells also displayed a Pasteur Effect since a fraction of glycolysis was indeed attenuated by oxygen, often to a similar extent as in normal growing cells [15].

Even though Warburg was also nominated for the Nobel Prize in 1926—for which he considered himself duly eligible—the Nobel committee decided to award it solely to Johannes Fibiger, honoring his findings on a gastric tissue growth condition believed to be a cancer induced by a nematode (spiroptera carcinoma). This has been considered as a misjudgment of the Nobel committee, as it later turned out not to be true [3, 16].

### The respiratory ferment

Along with the basic questions on what kind of changes in energy metabolism convert normal cells to tumor cells, Warburg was interested in the chemical basis for the “respiratory ferment” responsible for oxygen transfer in cells. Warburg had already postulated in 1914 that iron had a catalytic function in cellular respiration. Also, Warburg knew David Keilin, who (in 1925) had spectroscopically detected three cytochromes with iron-containing porphyrins (hemins) in respiring cells. Since the available amount of the ferment was too small for analytical chemistry, Warburg applied an “inhibition technique” by using two substances having specific inhibitory effects: hydrocyanic acid and carbon monoxide (CO), the first inhibiting respiration irreversibly, the latter reversibly depending on O_2_-pressure [17]. Visiting Warburg’s lab, Alan Hill brought to his attention that the inhibition of respiration by CO was light-sensitive. This allowed Warburg to characterize the oxygen-sensitive ferment by relating changes in the absorption coefficient determined by spectrometry with increases in the respiratory activity upon increasing the illumination [17]. In this way, Warburg identified *cytochrome a*_*3*_ (cytochrome oxidase) as being the CO-sensitive respiratory enzyme, i.e., the one requiring oxygen. Today, we know that there are indeed five proteins with iron for the electron transport and that cytochrome oxidase is part of complex IV. Warburg furthermore postulated the respiratory proteins to be localized in the “grana” of cells, which years later were identified as mitochondria.

## Reduction processes in metabolism

It was quite clear that if there were oxidative processes in life, there must also be reductive reactions, i.e., a transfer of hydrogens. From the beginning of the twentieth century, it was already known that some enzymes required hydrogen-mediated activation. While O_2_ was considered to be the physiological acceptor in the respiratory process, live cells were also able to reduce the non-physiological molecule methylene blue to a colorless compound in the presence of hydrogen donors such as succinate, malate, citrate, or glutamate. However, Warburg doubted that this synthetic methylene blue reduction reflected a physiological process until he visited E.S. Guzman Barron in his laboratory at the Johns Hopkins School of Medicine in Baltimore, USA, in 1929. They performed experiments with rabbit red blood cells (i.e., non-respiring cells), which were incubated with glucose and oxygen. The result was the production of pyruvate and CO_2_ along with the reduction of methylene blue [3]. Subsequently, Warburg sought for the chemical basis of this reaction. Having discovered that cell lysates incubated with phosphorylated glucose were also able to oxidize methylene blue, he now had an in vitro system for separating the components involved. Upon dialysis of the reaction solution, two essential molecular entities were discerned: (1) a heat-stable low molecular weight component, the coenzyme, and (2) a high molecular weight component, the enzyme. The latter actually contained a “yellow ferment”, which turned out to be a phospho-riboflavin, a nucleotide which is the prosthetic group of flavoproteins and can be reversibly hydrogenated and dehydrogenated.

As to the other low molecular compound, only a few milligrams had been isolated from red blood cells out of 200 l of horse blood. Three constituents of this molecule were soon identified as being phosphate, adenine, and a pentose; but one component remained elusive and its identification needed more material. As the anecdote from Hugo Theorell goes, Warburg (who loved and rode horses) had calculated that to have enough substance purified for further chemical analysis, he would need the blood from all the horses in Germany. Since Warburg was a very meticulous scientist in documenting all the measured physical and analytical parameters, his close friend Walther Schoeller (who worked for Schering on the “cancer problem”) suggested looking in the “Beilstein”, an encyclopedia of organic chemistry, for a corresponding compound. Indeed, there, he found it; it had already been synthesized in 1878 and was cheaply commercially available: nicotinamide [3, 18]. The complete molecule exists in either its phosphorylated or non-phosphorylated form, today known as NADPH and NADH, respectively, and is the catalytic active group for hydrogen transfers in about 150 known enzymes, i.e., dehydrogenases and NAD(P)H-oxidases. Moreover, Warburg hoped this coenzyme would have anti-cancer effects. This was not to be the case; but it was the basis for a medication against tuberculosis [4].

Together with the physicist Manfred von Ardenne, Warburg had improved the sensitivity of the spectrometer (1934/1935), which allowed him to measure the light absorbance spectra of these pyridine nucleotides. The discovery of differences in the spectral lines with the state of hydrogenation opened novel possibilities to measure the activity of enzyme reactions, which involved the transfer of molecular hydrogens from or to these pyridine nucleotides. This NADH resp. NADPH-linked analysis was the advent for measuring numerous enzyme activities, today known as the optical test. Indeed, over the next 10 years, using this assay, Warburg was the first to crystalize and characterize 9 of the 13 glycolytic enzymes already known by the reactions they catalyzed. Among them are lactate dehydrogenase, enolase, glyceraldehyde phosphate dehydrogenase (GAPDH), and pyruvate kinase—all key enzymes now again being studied in cancer metabolism. Further technical improvements on the photometer in cooperation with California-based Beckman Instruments made the device commercially available, and thereby this optical test became a world-wide enzymatic and analytical tool.

## The founding of the Institute of Cell Physiology in Berlin and world politics

Warburg deemed academic tourism a waste of time, but he did make two visits to the USA in the 1920s, visits which would have profound consequences on his career. On the first occasion in 1924, he visited Jacob Loeb, a friend of the Warburg family, at the Rockefeller Institute in New York, and gave talks also at other universities. In 1929, he was guest in the laboratory of Barron at Johns Hopkins Medical School, where he performed the experiments on the enzymatic nature of chemical reductions in blood cells. On this visit, with the support of Jacob Loeb, he also negotiated with the Rockefeller Foundation, who was interested in establishing research institutes in Germany. In 1930, the Rockefeller Foundation offered US$665,000 to the KWI for building two new institutes in Berlin: one for physics and one for cell physiology. The Institute of Cell Physiology was inaugurated in December 1931 and Otto Warburg became to be its one and only director.

The year 1931 was also the one in which Otto Warburg received the Nobel Prize in Medicine and Physiology for unraveling the oxygen-transferring ferment of respiration [17]. His father had missed this event by only a few months, having died in July.

A controversial and enigmatic issue is how Warburg could continue working in Berlin during the Third Reich, since his Jewish colleagues had departed or been expelled. Warburg himself was half-Jewish, but like his father considered himself to be a German Christian and even denied being related to the other Jewish descendants in the family. On the other hand, he was quite aware of the political situation from the very beginning and supported his Jewish coworkers in getting jobs outside of the country; he advised Hans Krebs to take a position in England. His rather disrespectful behavior toward a visiting governmental official jeopardized the institute being delivered with required chemicals. Moreover, in 1941, he was to be removed from his position as a director. However, probably mediated through indirect contacts to officials close to Hitler, he was allowed to keep this position and continue working in the institute. One explanation proposed for this salvation is that Hitler, having had a benign larynx polyp removed, was obsessed with cancer phobia and probably hoped for a rapid cure to be discovered. The institute was declared as a Service for Military Requirements (transliteration of “Wehrbetrieb”) with the mission to work on cancer therapies. In 1943, due to the bombings on Berlin, Warburg along with very few remaining coworkers had to move the laboratory to a small location in the countryside northwest of Berlin [4].

After the war, it took some years until Warburg could go back to acceptable working conditions. The Russians had confiscated the laboratory equipment. His institute in Dahlem (in West Berlin) was occupied by American troops. He was examined on his role in Nazi Germany. However, he finally was elected as a member of the Academy of Sciences (reestablished in East Berlin), of which his father along with Albert Einstein and Max Planck had already been members. Warburg had job offers from Russia, the USA, and from other countries and several German cities. Eventually, in 1948, he traveled to the USA, accepting the invitations from Robert Emerson at the University of Illinois and Dean Burk at the Cancer Institute at Bethesda to perform experiments on photosynthesis in their laboratories. In the summer of 1949, he also visited Woods Hole in Cape Cod and the Cancer Institute at Bethesda, where he met friends, expatriates, and critical colleagues, with anecdotal incidences of polemic scientific disputes. Returning to Berlin, he recovered “his” institute in 1950, which in 1952 was converted into a Max Planck institute, where he continued working until his death in 1970 [4].

## Concepts on tumor metabolism, the origin of cancer and its prevention

During the last period of his life, Warburg continued working on both photosynthesis and tumor metabolism, particularly on the role of respiration in tumorigenesis. For the latter, he now also used cultures of cells which had been isolated from normal tissues by trypsinization based on a method which had just been developed by Dulbecco and Vogt [19, 20]. Warburg reiterated his hypothesis on the cause of cancer, claiming that “the respiration of all cancer cells is damaged” in his famous Science paper [9]. Aspects of the controversial debate that followed are documented in a subsequent Science issue [21–23]; the disparate points of view appear to evolve from different understandings on defining the “damage” of mitochondria or the “chicken and the egg.” Moreover, Warburg published his ideas on the primary (ultimate) and secondary (distant) causes of cancer (transliteration of “Ueber die letzte Ursache und die entfernten Ursachen des Krebses”): while there are uncountable secondary causes, almost everything causing cancer, including time, the primary cause is the replacement of cell respiration by fermentation, i.e., lactate formation (quoted in [3]). He presented these concepts at prestigious meetings, one being the Annual Meeting of the Nobel Laureates in 1966 in Lindau, an island in the Lake of Constance (Germany). On this occasion, he received much scientific dissent. His idea that the causative problem of cancer cells was solely one of energetics and at the level of the respiratory system was not convincing in view of the fact that it contradicted Warburg’s own initial data and similar results from many others, and that it disregarded the more recent discoveries on the altered genetics of cancer cells leading to unregulated proliferation and tumor growth. It is inferred that Melvin Calvin, who had received the Nobel Prize on photosynthesis in 1961 and had expanded his interests to carcinogenesis, may have avoided these harsh confrontations with Warburg by not attending the Lindau meetings until 1974, after the death of Warburg [24].

Warburg claimed that 80 % of cancers could be avoided—simply by reducing the level of carcinogens [25]. Warburg considered cancer to be a nutritional problem, one that could be avoided by maintaining an appropriate natural diet. As early as 1923, Warburg and Schoeller discussed starving cancer by drugs leading to “nutritional deprivation” [4]. In his last publication in 1970, he claimed that a cause for spontaneous “tumor metabolism” was either a lack of oxygen or a lack of vitamin B1 (thiamin)—both conditions increasing the production of lactic acid [25]. This line of thinking led him to consider the administration of vitamin supplements, which would enhance respiration and was considered to be a natural and safe application. Already in the 1940s, Warburg, who was asserted having cancer phobia, practiced his own recommendations in maintaining a disciplined lifestyle: he grew his own vegetables, drew water from an unpolluted well, had his bread baked with grains from wheat not treated with pesticides, and kept his own poultry. And, he did sports: long walks, horseback riding, or sailing [3]. After his favorite sister Lotte died of cancer in 1948, Warburg also quit smoking [4]. Warburg, being sensitized to the dangers of smoking, alcohol, and drugs, proposed to the German Ministry of Health to reduce cigarette smoking, motor vehicle exhausts, air pollution, and chemical additives in foods as cancer prevention measures. This was in 1954 and at that time without avail [3].

## Conclusions

What are the effects of Warburg’s work and ideas on cancer research today? For one, there is the Warburg effect, i.e., the observation that tumors have a high rate of glycolysis, namely lactate production, in the presence of oxygen. While this is a reproducible observation, the scientific controversies continue on how the Warburg effect is related to the origin of cancer, what it means in molecular terms, how it can be connected to the genomics, proteomics, and molecular biology of cancer cells, and last but not least, how it can be utilized for diagnostics and therapy. The renaissance of the Warburg effect has put cancer metabolism back into the lime light of cancer research. Several recent reviews have drawn a bridge between Warburg’s original work and many aspects of molecular cancer metabolism and signaling pathways required for tumorigenesis known today (for example: [26–30]).

Probably the most controversial legacy for tumor metabolism is the hypothesis on the origin of cancer: that damaged respiration is solely responsible for the tumor type metabolism. In spite of its disaffirmation by numerous data and alternative explanations [31] providing evidence that there are also tumor cells which have apparently normal mitochondria and respiratory activity, what makes this hypothesis so attractive? A popular hypothesis always has two sides. The shiny side of the coin is the myriad of ideas it evokes and the new experiments and concepts it stimulates, especially when the hypothesis comes from a Nobel Laureate. On the other side, however, there is the danger of oversimplification and non-reflected universal application, as well as the uncritical acceptance of a *hypothesis* as a given fact. For the latter, Warburg alone cannot be held responsible—it is the challenge and responsibility of every scientist to question the validity of a hypothesis. We now have available many new investigative tools allowing us to elaborate on the observations and conclusions made by Otto Warburg about 90 years ago. He has set an example of meticulous work, bringing forth a gain of knowledge, which is now taken for granted as common textbook knowledge. He has, moreover, set an example for ingenious thinking, even if not all of it has been proven right. “Truth is more likely to come out of error if it is clear and definite, than out of confusion, and my experience teaches me that it is better to hold a understood and intelligible opinion, even if it should turn out to be wrong, than to be content with a muddled-headed mixture of conflicting views, sometimes called impartiality, and often no better than no opinion at all.” (Warburg quoted by Krebs, Schmid [3], p.83). Reflecting this statement could also be a Warburg effect.

## Abbreviations

KWI: Kaiser Wilhelm Institute
